# Effect of myostatin deletion on cardiac and microvascular function

**DOI:** 10.14814/phy2.13525

**Published:** 2017-11-30

**Authors:** Joshua T. Butcher, M. Irfan Ali, Merry W. Ma, Cameron G. McCarthy, Bianca N. Islam, Lauren G. Fox, James D. Mintz, Sebastian Larion, David J. Fulton, David W. Stepp

**Affiliations:** ^1^ Department of Pharmacology Department of Physiology Vascular Biology Center Augusta University Augusta Georgia

**Keywords:** Augmented muscle mass, cardiac function, coronary microvasculature, exercise, myostatin, nitric oxide, *β*‐adrenergic

## Abstract

The objective of this study is to test the hypothesis that increased muscle mass has positive effects on cardiovascular function. Specifically, we tested the hypothesis that increases in lean body mass caused by deletion of myostatin improves cardiac performance and vascular function. Echocardiography was used to quantify left ventricular function at baseline and after acute administration of propranolol and isoproterenol to assess *β*‐adrenergic reactivity. Additionally, resistance vessels in several beds were removed, cannulated, pressurized to 60 mmHg and reactivity to vasoactive stimuli was assessed. Hemodynamics were measured using in vivo radiotelemetry. Myostatin deletion results in increased fractional shortening at baseline. Additionally, arterioles in the coronary and muscular microcirculations are more sensitive to endothelial‐dependent dilation while nonmuscular beds or the aorta were unaffected. *β*‐adrenergic dilation was increased in both coronary and conduit arteries, suggesting a systemic effect of increased muscle mass on vascular function. Overall hemodynamics and physical characteristics (heart weight and size) remained unchanged. Myostatin deletion mimics in part the effects of exercise on cardiovascular function. It significantly increases lean muscle mass and results in muscle‐specific increases in endothelium‐dependent vasodilation. This suggests that increases in muscle mass may serve as a buffer against pathological states that specifically target cardiac function (heart failure), the *β*‐adrenergic system (age), and nitric oxide bio‐availability (atherosclerosis). Taken together, pharmacological inhibition of the myostatin pathway could prove an excellent mechanism by which the benefits of exercise can be conferred in patients that are unable to exercise.

## Introduction

The cardiovascular benefits of exercise are well characterized but a significant population in the United States is constrained by time, access, or preexisting conditions and unable to appropriately exercise, depriving them of these cardiometabolic improvements (Myers 2003; Lee et al. 2012; Arem et al. 2015; Carlson et al. 2015; Gebel et al. 2015). To address this, substantial effort has focused on understanding the key mechanisms that drive the cardiometabolic benefits of exercise, independent of physical activity (Matsakas and Diel 2005; Camporez et al. 2016). One important aspect of exercise is increases in muscle size. Myostatin, a member of the TGF‐*β* superfamily, has been identified as an important negative regulator of skeletal muscle mass (Matsakas and Diel 2005; Patel and Amthor 2005). Myostatin is a skeletal muscle myokine and its active form binds to the type 2B activin receptor on skeletal muscle, activating multiple pathways of muscle wasting gene transcription factors and decreasing phosphorylation of AKT (Han and Mitch 2011; Camporez et al. 2016). Myostatin is highly conserved across species and mice with myostatin deletion have significant increases in skeletal muscle mass (hyperplasia and hypertrophy) or subsequent overexpression results in loss of muscle mass (Lee and McPherron 2001; Reisz‐Porszasz et al. 2003). In humans, significant increases in myostatin are observed with chronic or acute muscle atrophy and muscle wasting accompanying HIV infection, while case studies identifying a loss‐of‐function mutation describes hypermuscularity (Gonzalez‐Cadavid et al. 1998; Reardon et al. 2001; Schuelke et al. 2004). Antibodies targeting myostatin in rodents are effective at reducing muscle wasting in a variety of disorders (cachexia, AIDS, CKD, muscle dystrophy), altering adiposity, increasing insulin sensitivity and decreasing inflammation (Latres et al. 2015; Camporez et al. 2016).

To date, however, the effect of deletion of myostatin and its associated hypermuscularity on the cardiovascular system of healthy animals has not been defined (Morissette et al. 2009; Rodgers et al. 2009). In this study we examined the function and anatomy of the cardiovascular system in myostatin KO mice.

Cardiac function was assessed in vivo using high‐resolution ultrasound and vascular function was assessed in vitro in five relevant vascular sites – two muscular, two nonmuscular and the aorta. Blood pressure and heart rate were assessed using radiotelemetry and basal metabolic function determined using baseline plasma chemistry. Taken together, these studies provide the first data defining the effects of myostatin‐deletion‐induced hypermuscularity on the cardiovascular system.

## Materials and Methods

All animals were used in accordance within the National Institutes for Health (NIH) guidelines for the Care and Use of Laboratory Animals. All experiments were approved by the Augusta University Institutional Animal Care and Use Committee. Wild‐type (ICR) mice were purchased from Jackson Laboratory and crossed onto the myostatin knockout mouse, which were obtained as a kind gift from Dr. Se‐Jin at Johns Hopkins. Male mice (aged 12–25 weeks) were used for the duration of the experiments.

Body composition was recorded using a Bruker minispec LF90 TD‐NMR analyzer. Visceral fat was obtained by collecting all of the observable white fat within the body cavity. All chemicals (Sigma Aldrich) and commercially available kits were stored and used according to written instructions. Cholesterol (Cholesterol, Total) and triglyceride (L‐Type Triglyceride M) kits were obtained from Wako Diagnostics, insulin (Mouse Insulin ELISA) and leptin (Mouse/Rat Leptin ELISA) from ALPCO and glucose (ALPHATRAK 2) from Andwin Scientific. After sacrifice, organs were removed, weighed, and placed in 10% formalin. Hematoxylin and eosin (H&E) stained cardiac sections were prepared. The myocyte area and perimeter calculated, using ImageJ in five separate locations of each slide.

In vivo cardiac function measurements using B‐mode ultrasonography were conducted with a Visual Sonics Vevo 2100. Physiologic assessment of left ventricular function occurred under isoflurane anesthesia. Baseline characteristics of cardiac function were obtained and followed by IP injection with *β*‐blockade (propranolol, 3 mg/kg) or *β*‐stimulation (isoproterenol, 3 mg/kg). Measurements were obtained 5 min subsequent to injection.

Ex vivo microvascular reactivity was assessed as described previously (Qiu et al. 2014). Briefly, arteries were isolated under an Olympus dissection scope, mounted and secured onto glass cannula, using 10–0 silk, pressurized to an intramural pressure of 60 mmHg in Kreb's buffer, and allowed to equilibrate. Vasomotor tone was quantified using a Living Systems small vessel arteriograph and pressure altered, using a Pressure Servo System PS\200. Subsequent to baseline equilibration, dose– response curves to the pharmacological stimuli shown in Figures 3–5 were obtained. Vessels were incubated in a calcium‐free environment to obtain passive wall mechanical measurements, as described previously (Stepp et al. 2004). Aortic vascular reactivity was obtained using wire myography (DMT). Endothelium‐dependent dilation was assessed using preconstriction to phenylephrine (1 *μ*mol/L), as previously published (Stepp et al. 2013). Dose–response curves to acetylcholine (10^−10^–10^−5^mol/L), sodium nitroprusside (SNP, 10^−10^–10^−4^mol/L), endothelin‐1 (ET‐1, 10^−11^–10^−7^mol/L), papaverine (10^−9^–10^−4^mol/L), and isoproterenol (10^−9^–10^−4^mol/L) were conducted. L‐NAME was used at a 500‐*μ*mol/L concentration. The following equation was used to calculate % dilation = (ID_(dose)_–BD_(active)_)/BD_(passive)_–BD_(active)_, where ID = inner diameter, BD = baseline diameter at active and passive measurements. Wall thickness was determined as the difference between passive inner and outer diameter at a specific pressure. Cross‐sectional area was calculated from the equation π*r* ^2^‐π(*r*‐IMT)^2^(Stepp et al. 2004).

In vivo hemodynamics were assessed with DSI PAC10 transmitters (blood pressure). Briefly, the animals were sedated with isoflurane anesthesia and the animal's left carotid isolated. The catheter was threaded from the carotid branch to the aorta arch and the battery back was tunneled above the animal's right shoulder to the flank. The animals were allowed to recover from the surgery for 7 days and then baseline recordings of heart rate, systolic, diastolic and mean arterial pressure, and activity were obtained continuously (10 sec/10 min) for 7 days. Baseline metabolic indices were obtained, using a Columbus Instrument Comprehensive Lab Animal Monitoring System (CLAMS).

All data are expressed as mean ± SEM. N represents the number of mice used in each experimental group and statistical significance was accepted at *P* < 0.05. Results were analyzed with an unpaired Student's t*‐*test or a repeated measures 2‐way ANOVA, using GraphPad Prism 5.01.

## Results

Baseline anatomic and metabolic characteristics are shown in Table 1. There was a significant reduction in visceral fat in the myostatin KO mice. NMR analysis also showed significant reductions in total body fat in myostatin KO mice, coupled with a significant increase in lean muscle. Heart weight remained unchanged between the two groups. Plasma characteristics showed a minor increase in fasting blood glucose in the myostatin KO mice, as well as plasma cholesterol. Insulin and triglycerides were similar between the groups but plasma leptin was significantly reduced with myostatin deletion. Metabolic analysis showed no difference in activity between groups, either voluntary activity with a wheel, baseline activity on an XYZ plane, or *V*O^2^. Activity from blood pressure (XY) also showed no differences between the two groups.

**Table 1:** Contains the baseline metabolic and plasma indices of the control ICR mice and myostatin KO mice

	Control	MyoKo
Anatomic indices		
Weight (g)	45.4 ± 1.0	46.3 ± 0.6
Visceral fat (g)	1.49 ± 0.2	0.226 ± 0.02^a^
Total body fat %	10.3 ± 1.3	4.75 ± 1.1^a^
Total body lean %	65.1 ± 1.2	71.2 ± 1.1^a^
Heart weight (mg)	192 ± 5.5	193 ± 2.9
HW/BW ratio	0.43 ± 0.01	0.42 ± 0.01
Plasma characteristics		
Fasting glucose (mg/dL)	114.4 ± 4.5	129.8 ± 3.3^a^
Insulin (*μ*/L)	0.38 ± 0.1	0.24 ± 0.1
Triglycerides (mg/dL)	93.8 ± 8.2	84.2 ± 7.7
Leptin (pg/mL)	2569 ± 360	1602 ± 261^a^
Cholesterol (mg/dL)	94.8 ± 3.9	131.8 ± 5.3^a^
Metabolic indices		
Voluntary activity (wheel count, 24 h)	960.0 ± 310	210 ± 67
Baseline activity (XZY plane, 24 h)	78578 ± 3908	60433 ± 8520
*V*0_2_ (mL/kg·h)	2902 ± 60.8	2758 ± 231.0
Activity (24 h, XY plane from BP)	4.8 ± 0.9	7.2 ± 1.5

*N* ≥ 8 for anatomic and plasma characteristics, *N* ≥ 4 for metabolic indices.

a *P* < 0.05 against control.

**Figure 1:**
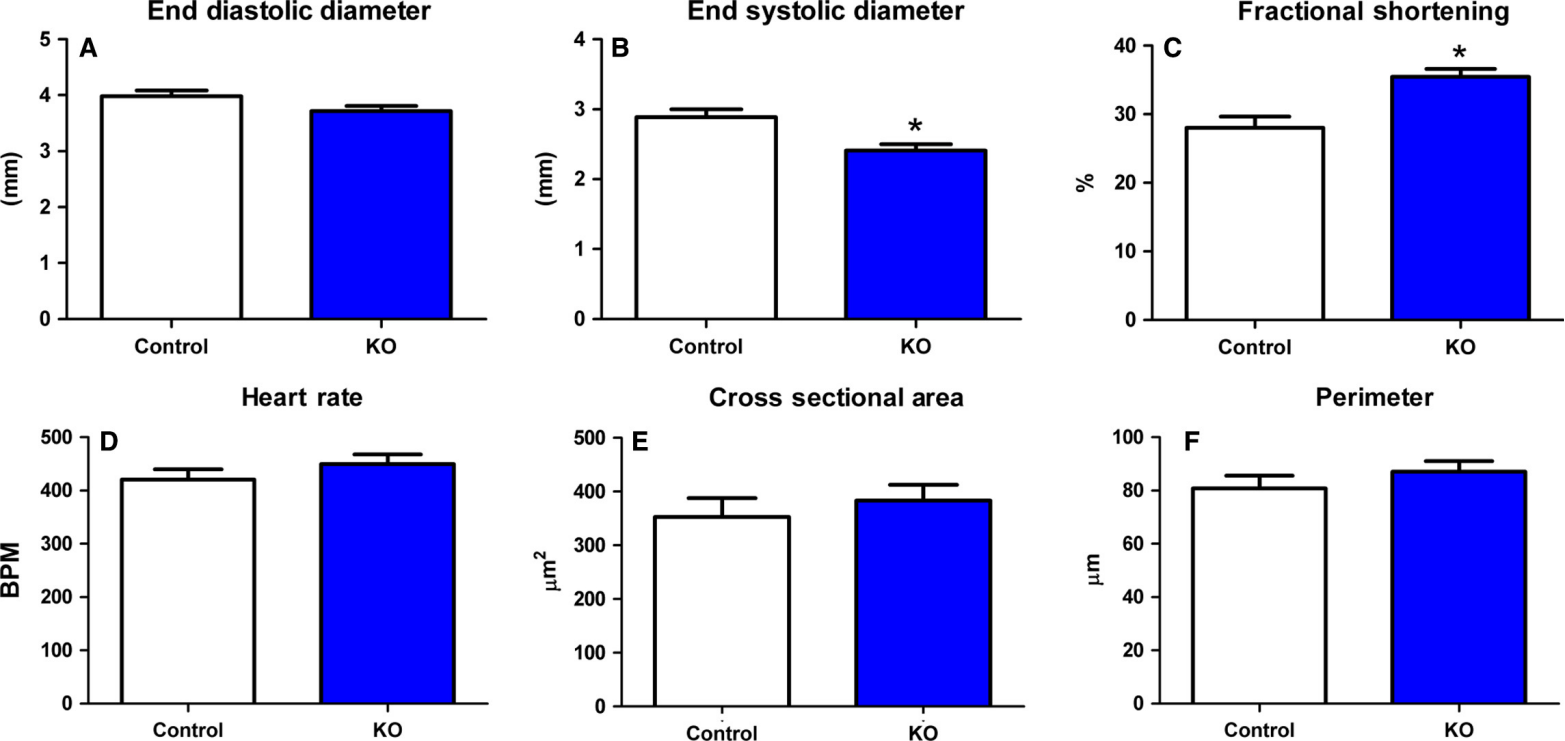
Baseline cardiac function is improved in myostatin KO mice. Ultrasound was used to examine baseline cardiac function in panels (A–D). End diastolic diameter (A) is unchanged between groups. End systolic diameter (B) is significantly reduced in the myostatin KO. Fractional shortening (C) is increased in the myostatin KO. Heart rate (D) is unchanged between the two groups. Panel (E & F) show myocyte characteristics. Myocyte size, assessed by cross sectional area (E) or perimeter (F) is unchanged between the two groups. *N* ≥ 7 and *P < 0.05 against control.

Figure 1 shows baseline cardiac function and characteristics in ICR controls (white) and myostatin KO mice (blue). Under basal conditions, there is no change in end diastolic diameter (Panel A). However, panel B shows a significant decrease in end systolic diameter and the resulting significant increase in fractional shortening (Panel C) in the myostatin KO mice compared to control. This increase in cardiac ejection fraction occurs independent of changes in heart rate (Panel D) or myocyte size (Panel E and F). Secondary parameters for these experiments are summarized in Table 2.

**Table 2:** Contains additional cardiac parameters obtained from echocardiography

Cardiac parameters	Baseline		Propranolol		Isoproterenol	
	Control	MyoKO	Control	MyoKO	Control	MyoKO
EDD (mm)	3.98 ± 0.10	3.72 ± 0.10	3.89 ± 0.18	4.01 ± 0.19	3.22 ± 0.09	3.12 ± 00.16
ESD (mm)	2.89 ± 0.50	2.41 ± 0.08^a^	3.20 ± 0.31	3.19 ± 0.22	2.02 ± 0.13	1.74 ± 0.16
Wall (D, mm)	1.1 ± 0.3	1.2 ± 0.4	1.1 ± 0.1	1.3 ± 0.1	1.6 ± 0.1	1.3 ± 0.1
Wall (S, mm)	1.4 ± 0.1	1.5 ± 0.1	1.3 ± 0.1	1.5 ± 0.1	2.0 ± 0.1	1.7 ± 0.1
Septum (D, mm)	0.9 ± 0.04	1.0 ± 0.03	0.9 ± 0.1	0.9 ± 0.04	1.1 ± 0.1	1.0 ± 0.05
Septum (S, mm)	1.3 ± 0.1	1.5 ± 0.05^a^	1.3 ± 0.1	1.3 ± 0.1	1.6 ± 0.1	1.6 ± 0.1
PWT (%)	31.8 ± 3.7	28.1 ± 3.0	16.5 ± 5.4	19.9 ± 6.5	26.3 ± 4.6	30.5 ± 4.7
HR(bpm)	420 ± 19	450 ± 18	367 ± 37	382 ± 31	448 ± 25	439 ± 24

EDD, End diastolic diameter; ESD, end systolic diameter; wall thickness at diastole (D) and systole (S), septal thickness at diastole (D) and systole (S) and posterior wall thickness % (PWT) of the left ventricle were obtained, as well as heart rate (HR) *N* ≥ 7.

a *P* < 0.05 against control.

**Figure 2:**
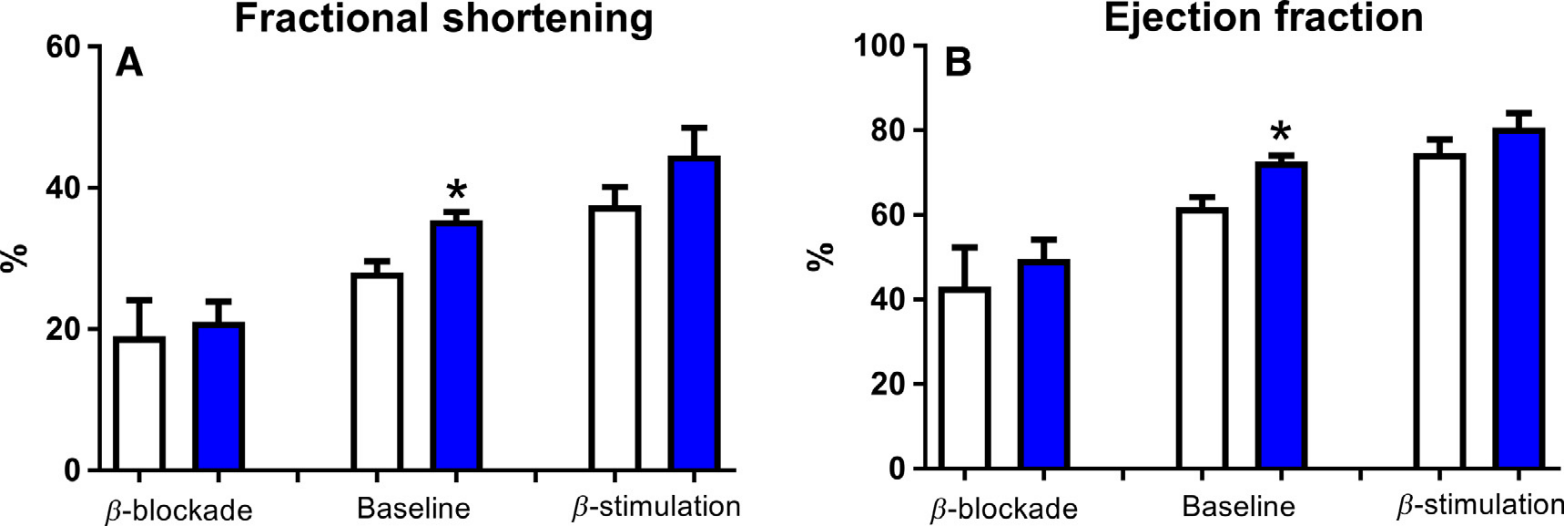
Effect of myostatin deletion on *β*‐adrenergic regulation of the heart. Ultrasound is used to examine *β*‐adrenergic adjustments to the heart. *β*‐blockade is accomplished using propranolol and *β*‐stimulation with isoproterenol. The increase in fractional shortening (A) and ejection fraction (B) at baseline is accompanied by parallel shifts down and up with *β*‐blockade and or antagonism, respectively, in each group. *N* ≥ 7 and *P < 0.05 against control.

Figure 2 summarizes the impact of *β*‐adrenergic tone to left ventricular cardiac function in control and KO mice. *β*‐blockade was accomplished with IP injection of propranolol and *β*‐stimulation with isoproterenol (Panel A and B). While baseline parameters are elevated as per Figure 1, when each respective genotype is fit to linear regression (inhibited, basal, stimulated), the slope of control (9.2 ± 0.1) was insignificant from the slope of the KO mouse (11.8 ± 1.5).

**Figure 3:**
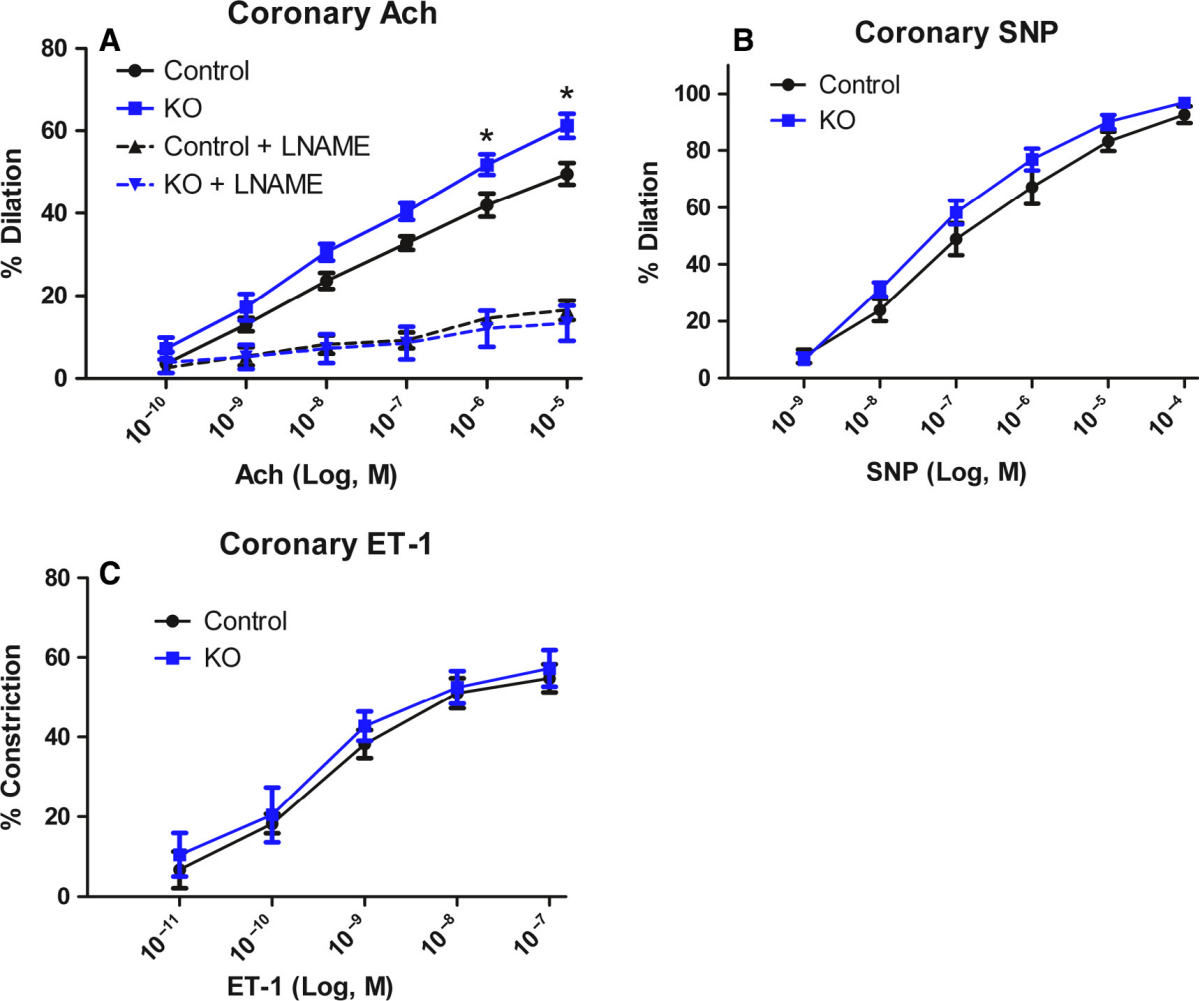
Comparison of endothelial modulation of vascular function. Myostatin KO mice have increased vasodilation to acetylcholine compared to control mice and this increase can be abolished with L‐NAME (A). Smooth muscle function, as assessed by SNP (B) or endothelin‐1 (C) was unchanged between the two groups. *N* ≥ 5 and *P < 0.05 against control.

Data comparing the endothelial and smooth muscle effects of myostatin deletion on coronary vasculature is shown in Figure 3. Panel A shows the dilator response of coronary arterioles to acetylcholine, an endothelial‐dependent dilator. Myostatin KO mice (blue line) show significant increases in dilator responses to increasing concentrations of acetylcholine compared to the control mice (black). Treatment with L‐NAME, a NOS inhibitor, blunted dilation in both groups and normalized the vasodilator response. However, dilator and constrictor responses to sodium nitroprusside (SNP, Panel B), an endothelial independent vasodilator, and endothelin‐1 (ET‐1, Panel B), a potent vasoconstrictor, were unchanged between the groups. Baseline inner diameter is significantly decreased in the control (49 ± 4 *μ*m vs. 71 ± 8 *μ*m), although baseline tone remained similar (38 ± 3% vs. 27 ± 5%).

**Figure 4:**
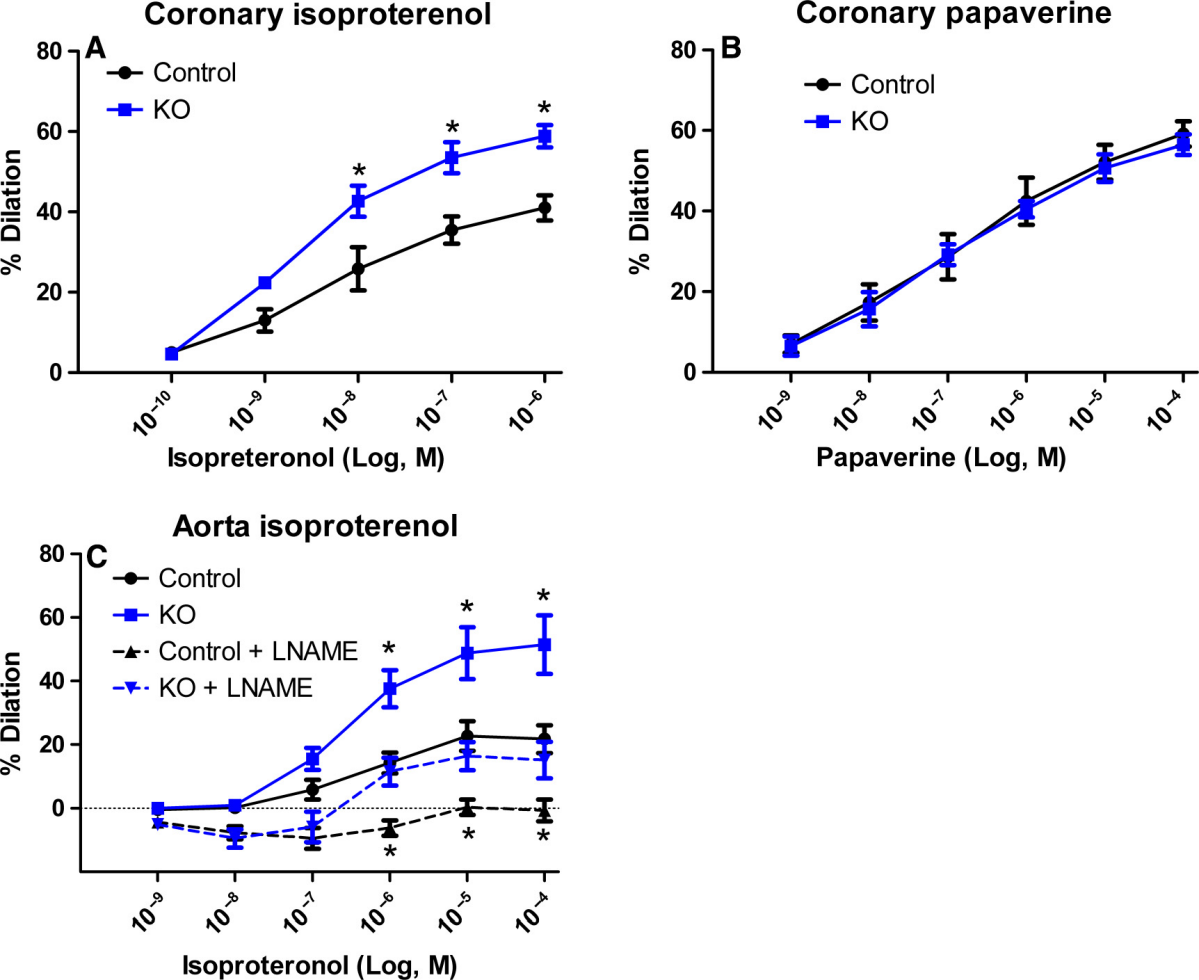
Further comparison of endothelial modulation of vascular function. Myostatin KO mice also have an increased vasodilatory response to isoproterenol (A). A dose– response curve to papaverine (B) shows no difference between the groups. Aortic reactivity to isoproterenol (C) shows a significant increase in dilation in the myostatin KO mouse and is blunted in the presence of L‐NAME. *N* ≥ 5 and *P < 0.05 against matched treatment control.

The effect of myostatin deletion on vasodilation is further characterized in Figure 4. Myostatin KO mice show a significant increase in dilation compared to control in response to isoproterenol (Panel A). The response to papaverine (Panel B), a compound used to transiently induce maximal coronary dilation, was unchanged between groups. In Panel C, reactivity to isoproterenol in the aorta showed a similar shift, suggesting that vascular *β*‐adrenergic sensitivity is a more ubiquitous effect of myostatin deletion. In response to L‐NAME, both groups exhibited parallel shifts downward, indicating a significant NO‐derived component. While the overall difference between the groups minimized, the myostatin KO group still exhibited significantly improved vasodilation to isoproterenol, suggesting additional alterations to the *β*‐adrenergic signaling axis.

Table 3 further describes the passive wall mechanics of the two groups. Myostatin deletion has no effect on incremental wall thickness, cross‐sectional wall area, wall:lumen ratio, or incremental distensibility at any pressure.

**Table 3:** Contains the passive wall mechanics at different pressures in the control and myostatin KO mice

Coronary parameters	Increment wall thickness		Cross‐section wall area		Walk lumen ration		Incremental distensibility (% mmhg)	
Pressure (mmHg)	Control	MyoKO	Control	MyoKO	Control	MyoKO	Control	MyoKO
20	22.5 ± 1.1	21.2 ± 1.3	6011 ± 1313	6836 ± 468	0.73 ± 0.06	0.53 ± 0.06		
40	20.3 ± 0.4	19.0 ± 1.6	5888 ± 234	6472 ± 510	0.57 ± 0.03	0.43 ± 0.06	1.55 ± 0.32	1.0 ± 0.23
60	18.8 ± 0.6	17.8 ± 1.9	5873 ± 254	6319 ± 553	0.48 ± 0.02	0.38 ± 0.06	0.99 ± 0.26	0.69 ± 0.12
80	17.4 ± 0.4	16.4 ± 2.0	5702 ± 249	6123 ± 619	0.40 ± 0.01	0.33 ± 0.06	0.98 ± 0.20	0.79 ± 0.12
100	16.1 ± 0.5	15.0 ± 2.0	5614 ± 271	5800 ± 669	0.34 ± 0.02	0.28 ± 0.05	0.92 ± 0.15	0.57 ± 0.06
120	14.3 ± 0.7	14.0 ± 2.3	5304 ± 270	5599 ± 755	0.28 ± 0.03	0.25 ± 0.6	0.98 ± 0.25	0.56 ± 0.08

*N* ≥ 5

No significant differences observed.

**Figure 5:**
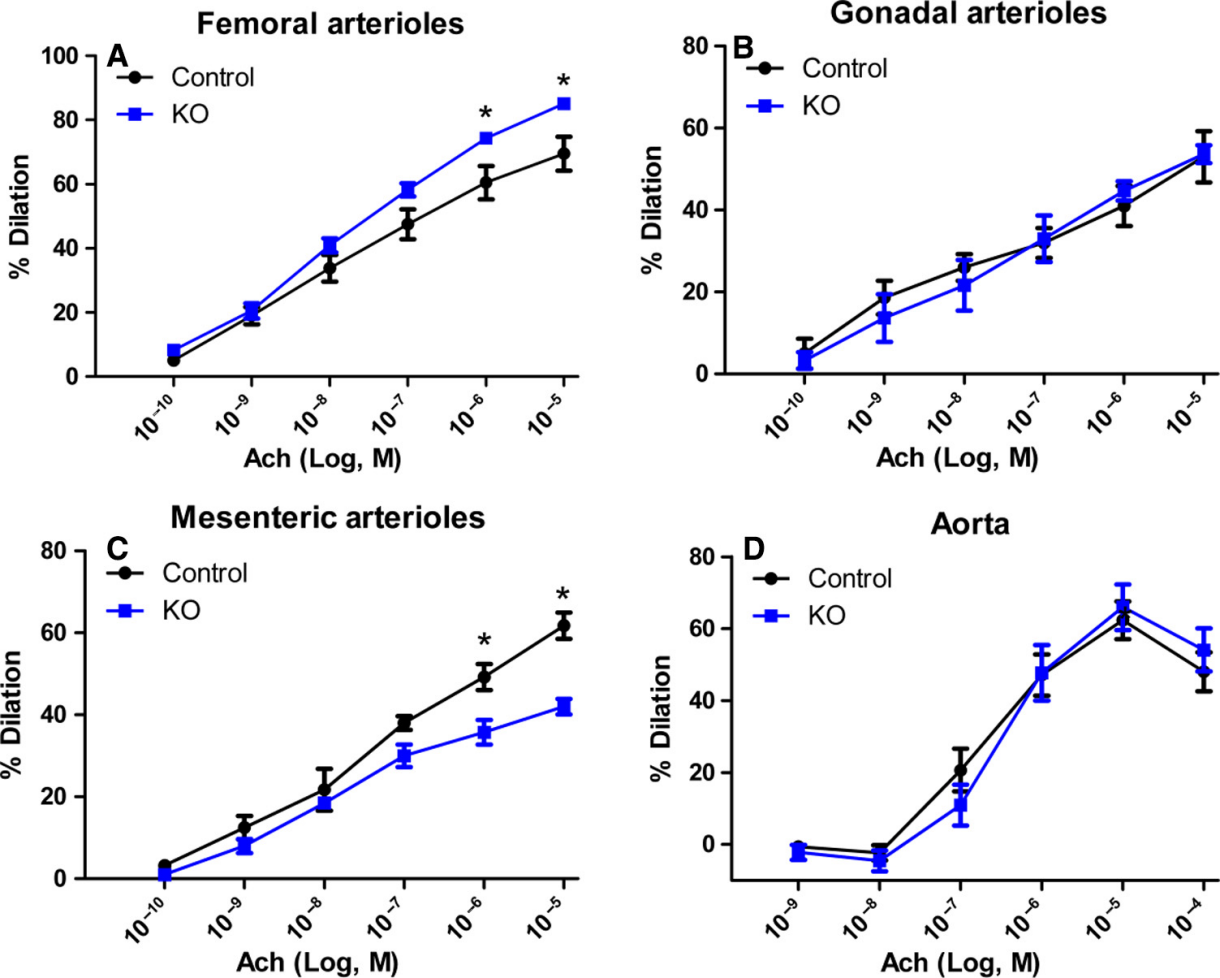
Myostatin deletion increases endothelial sensitivity in skeletal muscle resistance vasculature. Dose–response curves to acetylcholine in four different vascular beds are shown in Figure 5. Myostatin KO skeletal muscle vasculature (A) show a significant dilation in the myostatin KO mice compared to control. Gonadal arterioles (B) show no difference between the groups. Mesenteric arterioles (C) have a significantly blunted vasodilator response in the myostatin KO mice. The aorta (D) has no significant difference between the groups. *N* ≥ 8 for Panel A and *N* ≥ 3 for Panel B and C. *P < 0.05 against control.

To determine if the changes observed in the coronary circulation were typical or atypical, other vascular sites were assessed. Figure 5 shows the effect of myostatin deletion on nitric oxide bioavailability in four separate vascular beds. The femoral arterioles (A) of the myostatin KO mice have an increased vasodilatory response to acetylcholine compared to control, similar to what is observed in the cardiac vasculature. However, there was no increase in vasodilation observed in nonmuscular beds, including the gonadal arterioles (B), the mesenteric arterioles (C), and the aorta (D).

**Figure 6:**
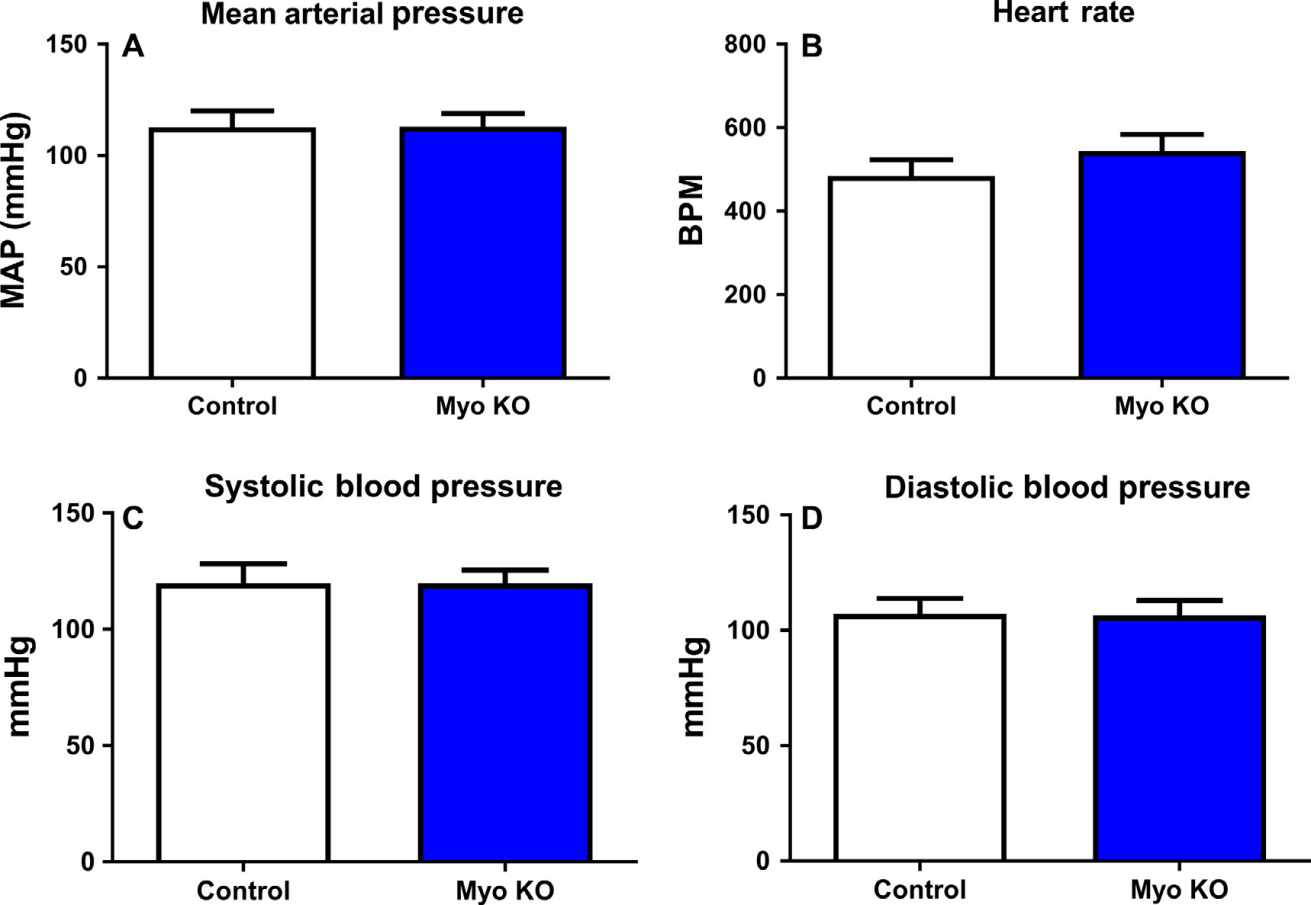
Myostatin deletion does not alter blood pressure or heart rate. Blood pressure (A),heart rate (B), systolic (C) and diastolic pressure (D) averaged over 7 days are unchanged between groups. *N* ≥ 5 and *P < 0.05 against control.

Figure 6 examines the effect of blood pressure regulation at baseline between two groups. Mean arterial pressure (Panel A), heart rate (Panel B), systolic (C) and diastolic (D) blood pressure remained unchanged between the two groups. Neither blood pressure nor heart rate differed between the myostatin KO and control.

## Discussion

Myostatin is a potent negative regulator of skeletal muscle growth and its therapeutic potential is of great interest in patients who are unable to adequately exercise. However, the global effect of myostatin deletion and the subsequent increase in muscle mass on cardiac function, vascular reactivity and blood pressure regulation in normal subjects remains incompletely understood. This study determined the effect of myostatin deletion on the cardiovascular system. The key findings are (1) that myostatin deletion produces a leaner animal with no major effects on baseline metabolism, (2) the deletion of myostatin increases fractional shortening of the heart and (3) there is an increase in vascular responsiveness, especially in muscular beds, to endothelial stimuli.

Exercise training is associated with multiple beneficial effects that have been well studied, often under pathological situations (heart failure, atherosclerosis, hypertension, etc.) (Hornig et al. 1996; Newcomer et al. 2011; Phillips et al. 2015; Adams et al. 2017). These benefits include increases in cardiac output, lower blood pressure, improved endothelial function, reductions in atherosclerotic lesions, and improvements in comorbidity and mortality (Niebauer and Cooke 1996; Hambrecht et al. 2000; Warburton et al. 2006; Dimeo et al. 2012; Green et al. 2017). However, these studies frequently focus on the effect of exercise in the context of a preexisting pathological condition although there are several studies that do demonstrate the importance of physical activity independent of changes in weight (Dengel 1985; Ross et al. 2000; Kraus et al. 2002; Cornelissen and Fagard 2005). Importantly the effects of individual aspects of the “exercise phenotype”, such as increased muscularity, remain largely unexplored in healthy individuals in terms of cardiovascular function. Our study sought to test the hypothesis that increased muscularity would impact cardiovascular function, using myostatin deletion as a tool to manipulate muscle mass, as would occur during exercise training in healthy individuals.

A cardinal benefit of exercise is augmentation of endothelium‐dependent vasodilation, with a large body of work implicating flow‐mediated dilation (shear stress) as a key regulator of exercised‐induced changes to vasoreactivity (Niebauer and Cooke 1996; Newcomer et al. 2011; Adams et al. 2017; Green et al. 2017). Coronary and skeletal muscle hemodynamics in humans, dogs and rodents have shown that exercise (both short and long term) has the effect of increasing blood flow in a nitric oxide‐dependent manner (Wang et al. 1993; Sessa et al. 1994; Koller et al. 1995). This study demonstrates a basal increase in endothelial cell sensitivity with myostatin deletion, an effect localized to muscular vascular beds (Figs. 3 and 5) – the heart and the hindlimb. This is demonstrated by similar increases in vasodilation to an endothelial‐dependent vasodilator (acetylcholine) in both femoral and coronary resistance vasculature. This effect is limited to skeletal muscle vasculature as gonadal and mesenteric arterioles, as well as the aorta, do not have increased sensitivity to endothelial‐dependent dilation and these changes occur independently of changes to vascular structure, smooth muscle cell constrictor behavior and responses to direct nitric oxide application. We could then infer that the mechanism of the improved vasodilation may relate to the heighted perfusion of these larger and presumably more metabolically active tissues.

Another key finding of our study is an increase in cardiac function as indicated by improved fractional shortening. During exercise increases in cardiac output occur to match perfusion with the increased metabolic demand. This study showed a basal increase in fractional shortening (Fig. 2) with myostatin deletion, which is consistent with results found with chronic exercise in humans (Ehsani et al. 1991). However, under *β*‐adjustments, the shifts in cardiac contractility were similar, suggesting an adaptation to increased preload as opposed to alterations to the *β*‐adrenergic signaling axis. Such an explanation would be consistent with improved dilation in muscular beds, diverting blood flow back to the heart to increase cardiac output. Additionally, given the lack of cardiac hypertrophy and changes in heart rate, this most likely is the response of the cardiac system to match perfusion with demand, a result of the increased lean body mass.

Finally, we observe a systemic augmentation of *β*‐adrenergic vasodilation. An increased propensity to dilate during periods of increased catecholamine release such as exercise would also confer a benefit to perfusion during states of heightened demand. The mechanism for this augmentation is not obvious but also relate to the metabolic state of individuals with changes in body composition caused by larger muscle mass. For example, a number of metabolic signals provide feedback control to the thyroid axis and thyroid hormones control *β*‐adrenergic reactivity (Parker et al. 1978; Tsujimoto et al. 1987; Nillni 2010). Whether hypermuscularity alters specific hormones in lean individuals remains to be determined. It is important to note that differences exist between this model of myostatin deletion and exercise. Namely, while both interventions would increase muscle mass, exercise has not been conclusively demonstrated to have a straightforward role in plasma glucose homeostasis and cholesterol, although suggested to improve, most likely due to varied exercise regimens, diets, and varied initial comorbidities and protocols (Yates et al. 2007; Cornelissen et al. 2011). However, myostatin deletion did modestly increase plasma glucose and cholesterol between our groups, although the increases are unlikely to exceed normal physiological baseline. Further myostatin deletion has been shown to contribute to muscle mass, specifically increased glycolytic fibers, but its effect on function remains unclear. Matsakas et al. (2012) showed that the muscle formed by germline myostatin deletion did not translate into more force generation, an effect not observed in other publications (Whittemore et al. 2003; Qiu et al. 2014). This current study did not address the specific muscle type or its quality, only that lean body mass is increased having an effect on baseline cardiovascular physiology. Additionally, the loss of fat with myostatin deletion has not been eliminated as an important contributor to vascular reactivity, although it is congruent with exercise models in rodents and humans (Dutheil et al. 2013; McMullan et al. 2016).

To summarize, our results suggest that increases in muscle mass can have positive effects on cardiovascular function. A higher lean body mass likely results in higher perfusion of muscular beds, shearing the endothelium and improving dilator function. The increased basal flow should, in turn, result in increased venous return to the heart to augment preload, increase cardiac function to support the higher perfusion at a normal pressure. We suggest that pharmaceutical manipulation of the myostatin pathway may confer these benefits that mimic, in part, the beneficial effects of exercise and protect against vascular disease.

## Conflict of Interest

None declared.
